# The differential role of *HTRA1* in HPV-positive and HPV-negative cervical cell line proliferation

**DOI:** 10.1186/s12885-016-2873-1

**Published:** 2016-11-03

**Authors:** Bruna Stuqui, André Luis Giacometti Conceição, Lara Termini, Laura Sichero, Luisa Lina Villa, Paula Rahal, Marília de Freitas Calmon

**Affiliations:** 1 Department of Biology, Instituto de Biociências, Letras e Ciências Exatas - IBILCE/UNESP, Rua Cristóvão Colombo n° 2265, Jardim Nazareth, CEP 15054-000 São José do Rio Preto, SP Brazil; 2 Center for Translational Investigation in Oncology, Instituto do Câncer do Estado de São Paulo, Hospital das Clínicas da Faculdade de Medicina da Universidade de São Paulo, Av. Dr. Arnaldo, 251, 8° andar, Bairro Cerqueira César, CEP 01246-000 São Paulo Brazil; 3 Department of Radiology and Oncology, Faculdade de Medicina, Universidade de São Paulo, Av. Dr. Arnaldo, 251, 8° andar, Bairro Cerqueira César, CEP 01246-000 São Paulo Brazil

**Keywords:** HTRA1, Cell proliferation, HPV, PDZ

## Abstract

**Background:**

High-risk human papillomaviruses (HPVs) are strongly associated with the development of some malignancies. The E6 and E7 viral oncoproteins are the primary proteins responsible for cell homeostasis alteration and immortalization. Furthermore, the E6 protein from high-risk HPVs can interact with the PDZ (PSD-90/Dlg/ZO-1) domains of cellular proteins, triggering cell transformation. One protein that is associated with pathological conditions and has a PDZ domain is the protease HTRA1 (high temperature requirement 1). This protein is poorly expressed in some cancers, suggesting a tumor suppressor role. The aim of this study was to evaluate the effect of HTRA1 overexpression in HPV16-positive (CasKi) and HPV-negative (C33) cervical cell lines.

**Methods:**

The cells were transfected with a vector containing the *HTRA1* ORF or an empty vector. *HTRA1* overexpression was confirmed by qRT-PCR. The cells were subjected to cell proliferation, colony formation, apoptosis and cell cycle assays.

**Results:**

C33 cells expressing HTRA1 grew significantly fewer colonies and showed less proliferation than cells without HTRA1 expression. In contrast, in the CasKi cells overexpressing HTRA1, there was an increase in the cell growth rate and in the colonies density compared to cells expressing low levels of HTRA1. An apoptosis assay showed that HTRA1 does not interfere with the apoptosis rate in these cells. A cell cycle immunofluorescence assay revealed more CasKi cells overexpressing HTRA1 in the S phase and more C33 *HTRA1*-transfected cells in the G0/G1 phase, suggesting that HTRA1 plays different roles in the cell cycle progression of these cells.

**Conclusions:**

HTRA1 overexpression prevents cell proliferation in the HPV-negative cell line and increases cell proliferation in the HPV-positive cell line. Although the E6/HTRA1 interaction has already been described in the literature, more studies are required to confirm whether the present functional findings are a result of this interaction.

## Background

High-risk human papillomaviruses (HPVs) are DNA viruses strongly implicated in the development of some malignances, such as cancer of the cervix (99 %) [1], anal canal (80–85 %) [2], vulva (40 %) [3], vagina (~70 %) [3], penis (~50 %) [4] and oropharynx (25 %) [5]. There are over 200 types of HPVs identified [6], but the malignant transformation of cervical epithelial cells is associated with persistent high-risk HPV infections, such as HPV 16 and 18 [7]. HPV 16 is responsible for up to 50 % of cervical cancers worldwide [8].

Cell pathways used by viral oncoproteins during viral replication are frequently disrupted, contributing to the development of HPV-associated cancers [9]. The E7 oncoprotein binds to the retinoblastome protein (pRb) and targets it for degradation, resulting in the release and activation of transcription factors (E2F) that drive S phase progression [10]. In the same way, the E6 oncoprotein binds to a cellular protein, E6-AP, and this complex interacts with p53, resulting in the ubiquitin-dependent degradation of the tumor suppressor p53 [11]. E6 is also responsible for regulating the transcription of some genes, for example, suppressing the expression of some tumor suppressors [12–14]. The E6 carboxy-terminal region conserved in high-risk HPVs is able to recognize and bind to human proteins containing PDZ (PSD-90/Dlg/ZO-1) domains, triggering their degradation, which increases E6 stability in infected cells [15, 16]. These cellular proteins are localized at the interfaces of cell-cell contacts and form signaling complexes that modulate cell growth, cell polarity, and cell adhesion [16–19]. The E6/cellular protein interactions are important to cancer progression induced by the virus [20].

The HTRA1 (high temperature requirement 1) protein is associated with several pathological conditions. This protease has a PDZ domain and is encoded by a gene located on chromosome 10 (10q26). Human HTRA1 belongs to a family of serine proteases involved in several important biological functions, such as protein quality control, cell growth, differentiation, apoptosis and degradation of extracellular matrix proteins [21, 22]. The HTRA1 protein contains an N-terminal regulatory domain, an insulin-like growth factor binding protease (IGFBP) domain, a trypsin-like serine protease domain and a C-terminal PDZ domain [23, 24]. The specific role of the PDZ domain of HTRA1 is not clear; however, it is known that PDZ recognizes C-terminal and internal hydrophobic sequences of target proteins, regulating HTRA1 protease activity [25, 26].

HTRA1 is involved in several pathologies [27–29] and some types of cancer [30–36]. This protease is expressed in several tissues, and transcription of its gene is highly regulated in both developing and adult tissues [24]. HTRA1 expression is decreased in some cancers, such as melanomas and lung and ovarian cancer, and the reduction of cell proliferation after an increase of its expression suggests a tumor suppressor role for this protein [31, 32, 36]. HTRA1 transcript downregulation was also observed in human keratinocytes immortalized with HPV16 compared to normal keratinocytes [37].

Although HTRA1 has been shown to interact with the E6 oncoprotein of high-risk HPVs, no studies have evaluated the role of HTRA1 in HPV-positive cells. Thus, in the present study, we investigated HTRA1 protein function in HPV-positive and HPV-negative cell lines.

## Methods

### Cell lines

HPV-16 positive (CasKi) (ATCC: CRL-1550) and HPV-negative (C33) (ATCC: HTB-31) human cervical carcinoma cell lines were grown in DMEM medium containing 10 % FBS (Fetal bovine serum) (Cultilab, SP, Brazil), supplemented with 100 U/ml penicillin (Invitrogen, Grand Island, NY, USA) and 100 μg/ml streptomycin (Invitrogen, Grand Island, NY, USA) and were grown in a 37 °C, 5 % CO_2_ atmosphere.

### Plasmids and transfection

pCMV6/Entry and pCMV6/HTRA1 were obtained from Origene Technologies (Origene Technologies, Rockville, MD, USA). pCMV6 vectors contain the neomycin phosphotransferase gene, which allows selection with a neomycin analog such as G418 (Sigma-Aldrich, St. Louis, MO, USA). The expression vectors were transfected into cell lines using Fugene HD (Promega, Madison, WI, USA) according to the manufacturer’s manual.

### Colony formation assay and cell growth curves

Forty-eight hours after transfection with pCMV6 or pCMV6/*HTRA1*, CasKi (9 × 10^4^) and C33 (6 × 10^4^) cells were trypsinized and plated in 6-well plates in media containing 800 μg/ml geneticin (G418, Sigma Aldrich, St Louis, MO, USA). The cells continued to grow for 14 days with media changes every 2 days; colonies were stained with 0.01 % crystal violet. Each experiment was performed in triplicate and in two independent assays.

To determine the cell growth rate, CasKi and C33 cells transfected and selected with G418 for 14 days were plated in 24-well plates (CasKi 3 × 10^4^ and C33 1 × 10^5^ cells). After 24, 48 and 72 h, the cell number was counted in a Neubauer Improved chamber.

### Apoptosis assay

Apoptotic cells were analyzed using a FITC Annexin V Apoptosis Detection Kit II (556570 - BD Biosciences, San Diego, CA, USA) according to the manufacturer’s instructions after they were transfected with pCMV6/*HTRA1* or empty vectors and subjected to 14 days of selection with geneticin. The cells were washed with PBS twice and then resuspended in binding buffer, and 5 μL FITC-Annexin V and 5 μL Propidium Iodide (PI) were added, after which the cells were incubated for 15 min in the dark at room temperature. The cells were analyzed using an easyCyte 5-HT flow cytometer (Millipore Guava Technologies, Hayward, USA). The data shown are from two independent experiments.

### Cell cycle analysis

After transfection and 14 days of selection with geneticin, the cell cycle was synchronized by the removal of FBS, and the cell cycle phases were assessed using the Cell Cycle Immunofluorescence Kit (558662 - BD Biosciences, San Diego, CA, USA). S phase cells were identified using BrdU and AlexaFluor 488 Mouse anti-BrdU, M phase cells were detected with an AlexaFluor 647 Rat anti-Histone H3 antibody (pS28) and G0/G1 phases were measured with DAPI, according to the manufacturer’s instructions. The cells were analyzed using an LSM 710 confocal microscope (Zeiss, Germany).

### RNA extraction and qRT-PCR

Total RNA was obtained using TRIzol reagent (Life Technologies, Grand Island, NY) according to the manufacturer’s instructions. Approximately 5 μg of total RNA from each sample were used to synthesize cDNA using the High Capacity cDNA Kit (Applied Biosystems, Foster City, CA, USA) according to the manufacturer’s instructions. Real-Time PCR was performed using an ABI Prism 7300 Real Time PCR system and SYBR Green PCR Core Reagent (Applied Biosystems, Warrington, UK) following the manufacturer’s protocol. The primer sequences were designed using Primer 3 software: *E6* HPV16 – GACCCAGAAAGTTACCACAG (Forward) and CATAAATCCCGAAAAGCAAAG (Reverse); *E7* HPV16 – ACAAGCAGAACCGGACAGAG (Forward) and TGCCCATTAACAGGTCTTCC (Reverse); *HTRA1* - CGCACTCATCAAAATTGACC (Forward) and CTGTGTTTTGAAGGGAAAACG (Reverse); *GAPDH* (endogenous control): ACCCACTCCTCCACCTTTGA (Forward) and CTGTTGCTGTAGCCAAATTCGT (Reverse). In brief, the reaction mixture (20 μL total volume) contained 25 ng of cDNA, gene-specific forward and reverse primers for each gene, and 10 μL of 2x Quantitative SYBR Green PCR Master Mix. The samples were tested in triplicate. The relative expression of each specific gene was calculated using the following formula: R = (E target)^∆Ct target (control - sample)^/(E endogenous)^∆Ct endogenous (control - sample)^, which was published previously [38]; a cutoff higher than a 2-fold change was used.

### Statistical analysis

Statistical analysis was performed using GraphPad Prism 5 Software. Functional comparisons between cells overexpressing *HTRA1* and cells with low *HTRA1* expression were performed using Student’s *t* test. In all analyses, the differences were considered statistically significant whenever *p* < 0.05.

## Results

### *HTRA1* overexpression in HPV-positive and HPV-negative cell lines

**Fig. 1:**
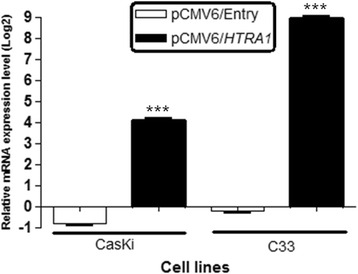
*HTRA1* overexpression in HPV-positive (CasKi) and HPV-negative (C33) cell lines. CasKi and C33 cells were transiently transfected with pCMV6/Entry (empty vector) or pCMV6/*HTRA1* and the overexpression of *HTRA1* was confirmed 48 h post-transfection by qRT-PCR. Quantitative mRNA expression of the *HTRA1* gene in both cell lines after transfection with pCMV6/*HTRA1* or the empty vector is shown as the fold change (log2) relative to expression

After transfection with the pCMV6/*HTRA1* expression vector or with an empty vector (pCMV6/Entry), *HTRA1* expression in the CasKi and C33 cell lines was accessed using qRT-PCR. The *HTRA1* gene was upregulated compared to cells transfected with the empty vector in both cell lines after transfection with the pCMV6/*HTRA1* vector (***p < 0.001) (Fig. 1).

### HTRA1 plays different roles in cell proliferation and colony formation in CasKi and C33 cell lines

**Fig. 2:**
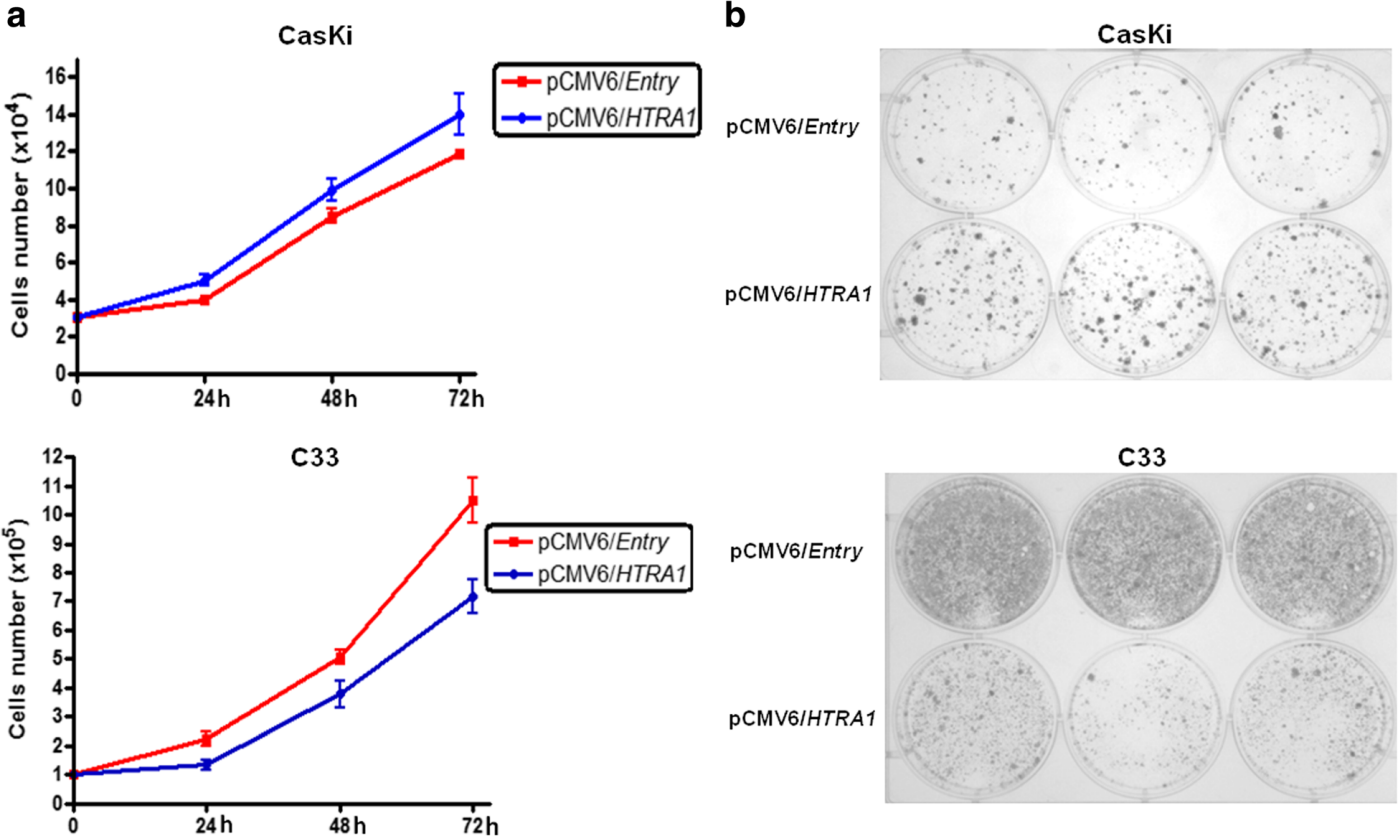
HTRA1 increases the proliferation and colony formation in CasKi cells and suppresses the same characteristics in the C33 cell line. Tumor cell proliferation was assessed *in vitro*. Cells were transiently transfected with pCMV6/Entry (empty vector) or pCMV6/*HTRA1* and replated 24 h post-transfection for selection with Geneticin/G418. After 14 days of selection, the cells were replated. **a** Growth curve analysis showed that the expression of HTRA1 increased cellular proliferation in CasKi cells and decreased cellular proliferation in C33 cells compared with that of the control cells (CasKi and C33 cells transfected with empty vector). **b** A colony formation assay showed a marked reduction in the number of colonies in the C33 cells expressing HTRA1 and an increase in the number of colonies in CasKi cells expressing HTRA1 compared to the control cells

Cell proliferation and colony formation ability were assessed after 14 days of selection of the transfected cells with G418. Our results demonstrate that CasKi cells expressing HTRA1 had an increased proliferation rate (Fig. 2a) and colonies density compared with the corresponding control cells (Fig. 2b). However, in C33 cells overexpressing HTRA1, a reduction in the cell growth rate (Fig. 2a) and colony number was observed compared to cells transfected with the empty vector (Fig. 2b).

### The apoptosis rate is not influenced by HTRA1

**Fig. 3:**
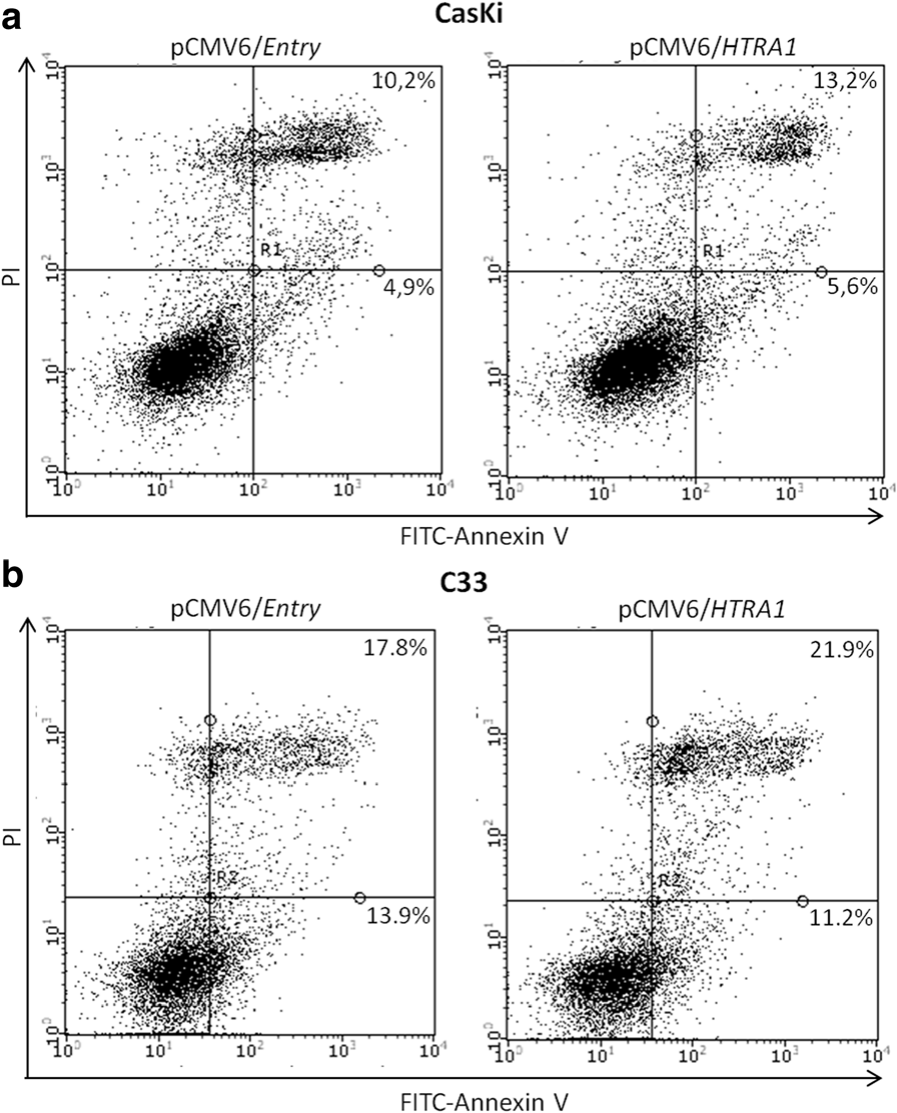
Effect of HTRA1 on apoptosis. Apoptosis in the CasKi (**a**) and C33 (**b**) cell lines was analyzed by flow cytometry after transfection and 14 days of G418 selection. No difference in apoptosis was observed in either cell line between cells overexpressing HTRA1 and those with low HTRA1 expression levels (*p* > 0.05). Viable cells are located in the bottom left (FITC-Annexin V negative/PI negative), early apoptotic cells in the bottom right (FITC-Annexin V positive/PI negative), late apoptotic or necrotic cells in the top right (FITC-Annexin V positive/PI positive) and necrotic cells in the top left quadrants (FITC-Annexin V negative/PI positive)

The ability of HTRA1 to induce apoptosis was also evaluated. The rate of apoptosis was assessed using FITC-Annexin V/PI after transfection with pCMV6/*HTRA1* and selection with G418. No significant difference in apoptosis was observed in both cell lines, whether overexpressing or underexpressing HTRA1 (*p* > 0.05) (Fig. 3).

### HTRA1 changes cell cycle progression

**Fig. 4:**
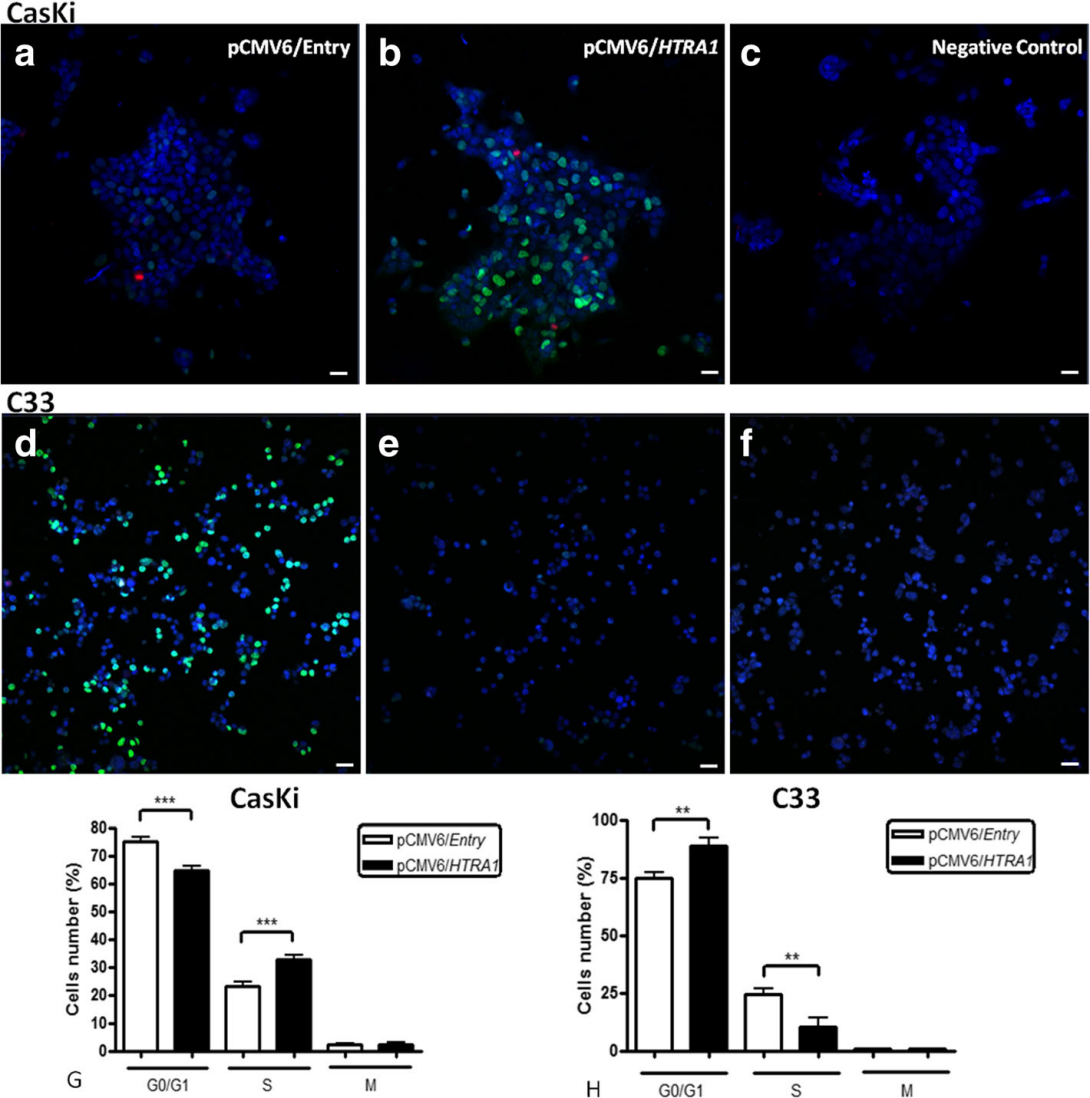
Effect of HTRA1 on the cell cycle in cell lines. Cell cycle phases in CasKi and C33 cell lines were analyzed post-transfection and after 14 days of G418 selection using an immunofluorescence assay. The number of CasKi cells in the S phase (*green*, AlexaFluor 488 Mouse anti-BrdU) and mitosis (*red*, AlexaFluor 647 Rat anti-Histone H3) was increased, while the number of cells in G0/G1 phase (*blue*) significantly decreased for cells overexpressing HTRA1 (**b**, **g**) compared with cells with low HTRA1 expression levels (**a**, **g**). The number of C33 cells overexpressing HTRA1 in G1/G0 was significantly increased (*blue*, Hoechst) and the number of cells in S phase was decreased (*green*) (**e**, **h**) compared to C33 cells without HTRA1 expression (**d**, **h**). **c** CasKi and **f** C33 cells incubated only with Hoechst were used as negative controls

Cell cycle analysis on HPV-positive and HPV-negative cells was performed using a cell cycle immunofluorescence assay after transfection with pCMV6/*HTRA1* and selection with G418. There were more CasKi cells overexpressing HTRA1 in the S phase (***p < 0.001) and fewer cells overexpressing HTRA1 in the G0/G1 phase after transfection (***p < 0.001) (Fig. 4a, b, c, g). The opposite was observed in the C33 cell line, in which a higher number of cells overexpressing HTRA1 was observed in the G0/G1 phase (**p < 0.01) than in C33 cells transfected with the empty vector (Fig. 4d, e, f, h).

## Discussion

In this study, we analyzed the effects of HTRA1 overexpression in HPV-positive (CasKi) and HPV-negative (C33) cell lines. Cervical carcinoma cells (C33) overexpressing HTRA1 had fewer colonies and a lower growth rate than the control. Studies using MTT assays have also reported that HTRA1 overexpression triggers a decrease in cell proliferation [22, 33], and colony numbers were reduced in soft agar assays [33]. Different investigations observed the downregulation of HTRA1 expression in various cancers types, such as melanoma, mesothelioma, lung, ovarian, bladder urothelial, breast, gallbladder and gastric cancer [31, 32, 36, 39–43]. Exogenous HTRA1 expression induces apoptosis and a reduction of cell proliferation in transformed cells, suggesting a tumor suppressor role for this protein [36, 44].

Xia et al. [45] showed a reduction in cell proliferation and invasion in esophageal squamous cell carcinoma overexpressing HTRA1 due to blockage of the nuclear factor-κB signaling pathway coupled to a decrease in the Ki-67, Bcl-2 (B-cell lymphoma 2), Bcl-xL (B-cell lymphoma-extra large), cyclin D1 and MMP-9 (matrix metalloproteinase 9) proteins. In endometrial cancer cell lines, exogenous HTRA1 expression resulted in a decrease in the invasive and migration potential of these cells. Thereby, the loss of HTRA1 may contribute to the aggressiveness and metastatic phenotype of cancer cells [35].

In contrast to that observed in C33 cells, in the HPV-16-positive cervical carcinoma cell line (CasKi) HTRA1-overexpressing cells showed an increase in colony formation and cell proliferation. This report is the first to describe the effect of HTRA1 overexpression in cells containing high-risk HPV. The increase in the colony number and cell growth rate in HPV-positive *HTRA1*-transfected cells could be explained by the viral replicative cycle characteristics. These viruses express early proteins - E6, E7 and E5 - that interact with cellular proteins and interfere with normal cell cycle regulation. The E6 oncoprotein of high-risk HPVs is able to interact with the PDZ domain of cellular proteins, preventing apoptosis and stimulating the proliferation of infected cells [46, 47]. The HTRA1 serine protease contains a PDZ domain in its C-terminal region, and for this reason, it is a strong candidate to interact with the E6 protein of high-risk HPVs [25, 26], and the association of both proteins may result in the bypass of growth arrest. In fact, interaction between HTRA1 and HPV E6 proteins was observed by Clawson et al. [48], however there are no functional studies that describe the effects of this association.

Some studies suggest that the interaction between the E6 PBM (PDZ domain-binding motif) and the PDZ domains of cellular proteins increases E6 stability, promoting high levels of E6 and HPV genome maintenance in the cell [16, 17]. Nicolaides [16] showed that the interaction between E6 and the PDZ domain of two cellular proteins involved in cell polarity, MAGI (Membrane Associated Guanylate kinase Inverted) and hScrib (Scribble Homolog Protein), increases E6 levels in immortalized keratinocytes (NIKS), probably by preventing them from proteasomal degradation. Furthermore, NIKS cells transfected with an HPV 16 genome mutated in the E6 carboxy-terminal region presented low levels of E6, and the viral genome was unable to remain as an epissome, becoming degraded or integrated into the host genome. For HPV 31, the loss of the E6 PBM domain was shown to trigger reduction in the viral copy number in human foreskin keratinocytes (HFK) [17].

We speculate that in CasKi cell line, E6 oncoprotein could interact with HTRA1 PDZ domain, triggering high levels of this viral oncoprotein in the cell and enhancing E6 cellular transformation activity, prompting the proliferation of HPV infected cells, which could explain the increased cell proliferation observed in HPV-positive cells overexpressing HTRA1.

The cell cycle was assessed in this study by immunofluorescence to determine whether the changes in cellular proliferation induced by HTRA1 overexpression could be explained by modifications in the proportion of cells in each cell cycle phase. In the HPV-negative cell line, HTRA1 overexpression triggered cell cycle arrest, increasing the number of cells in G0/G1 phase and reducing the number of cells in synthesis (S) phase. In the HPV-positive cell line, HTRA1 overexpression triggered cell cycle progression by increasing the number of cells in S phase and decreasing the number of cells in G0/G1. These results are in agreement with the observed decrease in the colony and cell numbers in C33 cells and increase in colony and cell numbers in the CasKi cell line after HTRA1 overexpression. The data suggest a tumor suppressor role only in HPV-negative cells (C33 cells) and an opposite effect in HPV-positive cells (CasKi cells).

One limitation of our study is that due to the different genetic background of the transformed C33 and CasKi cell lines, our experiments do not allow us to infer the mechanism by which HTRA1 overexpression reduced proliferation only in the HPV-negative cell line. However, it is possible that the interaction between E6 and HTRA1 showed by immunoprecipitation [48] depends on the PDZ domain and triggers a high level of E6 oncoprotein in the cell, enhancing its cellular transformation activity. However, other studies are necessary to investigate how HTRA1 increases cell proliferation in the CasKi cell line and in other HPV-positive cell lines and tumors.

## Conclusions

HTRA1 overexpression prevents cell proliferation in the HPV-negative cell line, as described in the literature in other tumor cells, via cell cycle arrest in G0/G1. On the other hand, HTRA1 increases cell proliferation in the HPV-positive cell line, inducing cell cycle progression by increasing the number of cells in S phase and decreasing the number of cells in G0/G1. However, more studies are required to determine whether this high rate of cellular proliferation is a result of the E6/HTRA1 PDZ interaction.

## Abbreviations

Bcl-2: B-cell lymphoma 2
Bcl-xL: B-cell lymphoma-extra large
FBS: Fetal bovine serum
G418: Geneticin
HFK: Human foreskin keratinocytes
HPV: High-risk human papillomaviruses
hScrib: Scribble homolog protein
HTRA1: High temperature requirement 1
IGFBP: Insulin-like growth factor binding protease domain
MAGI: Membrane Associated Guanylate kinase Inverted
MMP-9: Matrix metalloproteinase 9
NIKS: Immortalized keratinocytes
PBM: PDZ domain-binding motif
PDZ: PSD-90/Dlg/ZO-1 domain
pRb: Retinoblastome protein
